# Robotic total hip and knee arthroplasty: economic impact and workflow efficiency

**DOI:** 10.1007/s11701-025-02698-3

**Published:** 2025-09-08

**Authors:** Benjamin E. Jevnikar, Shujaa T. Khan, Ahmed K. Emara, Khaled A. Elmenawi, Matthew Deren, Nicolas S. Piuzzi

**Affiliations:** Department of Orthopedic Surgery, Orthopedic and Rheumatology Institute, Cleveland Clinic Foundation, 9500 Euclid Ave, Cleveland, OH A4144195 USA

**Keywords:** Arthroplasty, Replacement, Knee, Hip, Robotics, Health care costs

## Abstract

Robotic-assisted total joint arthroplasty (RA-TJA) is projected to account for 70% of all arthroplasties by 2030, yet its economic value and operational efficiency have yet to be thoroughly synthesized. While early literature emphasized technical precision, evolving payment models and implementation costs have shifted focus toward cost-effectiveness and workflow integration. To evaluate the economic and institutional viability of RA-TJA by synthesizing available evidence on capital costs, perioperative expenses, learning curves, throughput, and long-term adoption trends. This review also considers market competition, global uptake, and the need for standardized outcomes reporting. A narrative literature review of published cost analyses, real-world efficiency studies, and policy-relevant frameworks was conducted. Literature addressing per-case cost variability, bundled payments, and implementation strategies was analyzed. Emerging economic models and global diffusion patterns were incorporated to contextualize long-term feasibility. Robotic platforms consistently incur higher upfront and perioperative costs than manual or navigated arthroplasty. However, in both high- and low-volume institutions, these costs may be offset by reduced complications, shorter hospital stays, and improved discharge metrics. Efficiency gains are amplified through procedural clustering and experienced teams. Adoption is accelerating globally, facilitated by leasing models and market competition, yet value remains highly dependent on institutional context. A lack of standardized outcome reporting and platform heterogeneity limits cross-study comparisons. Ongoing, long-term, multicenter randomized trials are expected to address these evidence gaps. Robotic assistance can improve the efficiency and precision of arthroplasty procedures, with the potential for substantial cost-effectiveness when optimized. Institutions adopting this technology can expect improved outcomes by leveraging local surgical volume and infrastructure. Wider integration will be facilitated by robust long-term data, standardized outcome metrics, and continuous innovation that aligns with value-based care models.

## Introduction

Robotic-assisted total hip and knee arthroplasty (RA-THA and RA-TKA) have gained increasing attention in recent years as potential tools to enhance surgical planning, execution, and consistency. Although the initial introduction of robotic systems was met with hesitancy primarily due to substantial capital costs, increased operative times, and training requirements, adoption rates have risen sharply. Between 2015 and 2020, RA-TKA volume increased more than sixfold, and recent projections suggest that robotic systems could be utilized in up to 70% of all TKA and THA procedures in the United States by 2030 [1–4].

Proponents of robotic platforms cite potential advantages including improved component alignment, more personalized preoperative planning, and reduced variability in surgical technique [5, 6]. Yet, definitive clinical superiority over manual approaches remains a subject of ongoing debate, with studies reporting mixed outcomes depending on the platform, procedure, and setting. As enthusiasm grows, it is essential to assess the value of RA-THA and RA-TKA through the perspectives of economics and efficiency, domains that are often decisive in shaping institutional decisions and policies.

This review explores the economic and workflow implications of robotic-assisted total joint arthroplasty (RA-TJA), including initial capital investment, cost-effectiveness across different practice settings, intraoperative efficiency, and institutional integration. We also consider broader economic frameworks such as Moore’s Law and economies of scale to contextualize how cost barriers may shift as technology matures. By synthesizing the current evidence and practical considerations, we aim to provide a balanced perspective on the evolving role of robotics in primary THA and TKA.

## Economic overview of robotic arthroplasty

### Upfront capital and maintenance costs

The upfront financial investment required for RA-TJA remains one of the most substantial barriers to widespread adoption. Reported purchase prices for robotic systems typically range from $500,000 to over $1 million per unit, depending on the platform, included instrumentation, and vendor-specific service contracts [7–9]. These capital costs are frequently accompanied by annual maintenance fees ranging from $40,000 to $150,000, which are essential for preserving hardware functionality, ensuring software updates, and maintaining system warranties [7, 10]. In addition to these fixed costs, robot-specific disposable items, including cutting guides, drapes, and burrs, add several hundred to over $1,000 per procedure, further increasing the per-case expense relative to manual or computer-navigated approaches [8, 9]. These additive costs contribute directly to the elevated index surgery charges associated with RA-TJA, even before accounting for downstream care or potential savings [3, 11, 12].

Despite these substantial initial costs, advocates of robotic technology emphasize potential long-term benefits, such as more consistent component positioning and improved preoperative planning, that may reduce complications, revisions, and associated downstream costs [7, 13, 14]. However, the current literature consistently shows that RA-THA and RA-TKA are associated with higher upfront and maintenance costs than manual or computer-navigated approaches [3, 8, 9, 15, 16].

### Perioperative and episode-of-care costs

While much attention has been given to the capital acquisition and maintenance costs of robotic systems, their financial impacts across the entire episode of care must be considered. Several studies suggest that downstream savings, including as shorter length of stay**,** reduced post-acute care utilization, and lower complication rates, may partially or fully offset higher index costs, particularly in high-volume institutions [8, 10, 15, 17]. For instance, in a national Medicare analysis of over 198,000 patients undergoing TKA, Shah et al. found that for TKA, particularly in high-volume institutions, 90-day episode-of-care spending was $587 lower for patients who underwent RA-TKA compared to conventional TKA ($13,676 vs $14,263; 95% CI: -$798 to -$375) [17]. This reduction in total cost was driven by several key factors: patients in the RA-TKA group had a shorter mean length of stay (1.9 vs 2.3 days), fewer complications (2.7% vs 3.3%), and were nearly twice as likely to be discharged directly home rather than to a post-acute care facility (16.8% vs 32.4%) [17]. Similar trends have been observed for RA-THA: robotic procedures incur higher initial costs than manual techniques but may offer advantages in terms of reduced dislocations**,** shorter length of stay, and potential reductions in reoperations [3, 8, 18].

In summary, while RA-TJA is associated with greater perioperative and episode-of-care costs than manual and computer-navigated approaches, these costs may be offset in high-volume centers through reduced complications, shorter hospital stays, and improved long-term outcomes. As bundled care models continue to evolve, institutional volume and care standardization will likely remain key determinants of cost-effectiveness.

### Site of care delivery

The site of care delivery is an important determinant of episode-of-care costs for RA-TJA. Across both Medicare and international health systems, ambulatory surgical centers (ASCs) consistently demonstrate lower overall costs compared with inpatient hospital admissions and hospital outpatient departments (HOPDs), while maintaining equivalent safety and clinical outcomes in appropriately selected patients. Recent analyses of Medicare Part A and B claims estimate annual savings of approximately $235 million when TJA is performed in ASCs rather than inpatient hospitals, and $137 million compared to HOPDs, with adjusted costs for primary total hip and knee arthroplasty averaging roughly $8,500 per case in the ASC setting [19]. Similar trends are observed in Canadian data, where outpatient arthroplasty, including robotic and manual, achieves perioperative and long-term savings of $3,800–$7,000 per case over a three-year period without increased adverse events [20].

While robotic-assisted techniques incur initially higher supply and capital costs relative to manual approaches, these differences are attenuated when accounting for patient factors, comorbidity burden, and facility efficiencies. In ASCs, RA-TKA and RA-THA have demonstrated safety profiles comparable to both conventional ASC procedures and hospital-based RA-TJA, with no increase in complication or readmission rates [21, 22]. Overall, shifting RA-TJA to the ASC environment can magnify the cost advantages inherent to outpatient care, provided that patient selection criteria, perioperative protocols, and facility resources are optimized. However, the incremental cost of robotic technology may offset some of these savings, underscoring the importance of aligning case selection, surgeon experience, and reimbursement structures to realize the full economic benefit [23].

### Value in high- vs. low-volume centers

One of the most influential determinants of cost-effectiveness in RA-TJA is institutional case volume. High-volume centers are positioned to extract greater value from robotic platforms by amortizing the substantial upfront capital and recurring maintenance costs across a larger number of procedures, while lower volume settings can still observe benefits of robotic implementation via reductions in episode-of-care costs.

Cost modeling studies have identified case thresholds of 24 to 50 RA-TKA procedures annually as the minimum required to achieve financial neutrality, meaning the point at which robotic assistance becomes economically comparable to manual techniques. Above this threshold, the economic benefits increase markedly. In centers performing more than 100–200 RA-TKA procedures per year**,** per-case costs can decline to approximately $3,900–$7,500, and incremental cost-effectiveness ratios (ICERs) as low as $2,331 per quality-adjusted life year (QALY) have been reported- well below standard willingness-to-pay benchmarks [10, 15, 17]. In these settings, RA-TJA may be not only cost-effective, but even cost-saving, especially when potential reductions in revision rates and long-term complications are considered [10, 15]. Importantly, high-volume centers also demonstrate superior clinical outcomes in arthroplasty overall, including lower complication rates**,** shorter length of stay, and reduced total charges, further enhancing the value proposition of robotic platforms [24]. These benefits, in combination with favorable economic indicators, reinforce the notion that robotic technology is best deployed in settings with the infrastructure and case throughput to optimize its potential.

While high-volume centers are more likely to recoup the upfront costs of robotic platforms through surgical throughput, emerging evidence suggests that RA-TJA may also offer economic advantages in lower-volume settings by reducing the incidence and downstream costs of perioperative complications. Multiple studies have demonstrated that RA-TJA is associated with lower rates of mechanical complications, infections, thromboembolic events, and transfusions compared to manual surgery, all of which are key drivers of episode-of-care costs [3, 8, 25–28]. Cotter et al. reported that even in a moderate-volume, single-surgeon practice, the higher intraoperative costs of RA-TKA were offset by significantly lower 90-day costs due to shorter length of stay and decreased post-acute care utilization, driven largely by reduced complication rates [8]. Similar findings were reported by Constantinescu et al. and Aggarwal et al., who observed fewer perioperative complications and shorter hospitalizations following RA-TKA [25, 27]. Thus, even in smaller programs, robotic systems may provide value by mitigating the costly burden of complications commonly associated with conventional instrumentation.

### Medicare vs private payer implications

The cost-effectiveness of RA-TJA is not only shaped by institutional volume and system costs, but also by the type of payer and associated reimbursement structures. In particular, Medicare and private insurers differ substantially in how surgical episodes are reimbursed, which affects a hospital's ability to recover the upfront and perioperative costs associated with robotic platforms.

Within the Medicare system, reimbursement is fixed and standardized, which can constrain cost-effectiveness in lower-volume centers. However, high-volume institutions may achieve savings, particularly under value-based programs like the Bundled Payment for Care Improvement (BPCI) initiative. This is supported by numerous literature demonstrating that hospitals utilizing robotics for > 50% of their total arthroplasty volume have lower episode costs, shorter lengths of stay, and higher rates of discharge to home, all of which contribute to improved cost-effectiveness under bundled payment models [15, 17, 29, 30].

Yet, the broader financial trend of declining reimbursements in TJA complicates robotic viability in the Medicare population. A longitudinal study by Wang et al. revealed that from 2010 to 2018, Medicare surgeon reimbursements for TKA and THA declined significantly, falling 2 to 2.8 times faster than payments from commercial payers after adjusting for inflation [31]. On average, Medicare reimbursed 57% less than commercial payers for inpatient orthopedic surgeries, and Medicare payments per work relative value unit (wRVU) dropped notably for TKA (–0.83%) and THA (− 0.80%) over the study period [2]. These declining surgeon reimbursements may strain the financial feasibility of adopting high-cost technologies like robotic systems, particularly in practices with a large proportion of Medicare patients.

Both payer structure and payment trajectory play critical roles in determining the cost-effectiveness of robotic platforms. Medicare’s declining reimbursement rates and bundled payment pressures favor high-volume, highly efficient systems. As reimbursement for orthopedic procedures continues to decrease, particularly from Medicare, the bar for sustainable robotic adoption is likely to rise, reinforcing the importance of volume, care coordination, and outcome optimization.

## Efficiency and workflow impact

### Operative time and learning curve

A brief learning curve for RA-TJA is well documented in the literature. While robotic procedures are associated with slightly longer operative times of 12 to 25 min during early adoption, this modest increase is transient and typically normalizes after 6 to 20 cases, depending on the surgeon’s experience and the robotic system used [32–34]. In high-volume surgical environments, this early inefficiency has been shown to have negligible impact on patient outcomes or institutional costs [11, 16, 32, 35].

Importantly, the learning curve associated with robotic adoption is confined to operative efficiency and team coordination, not surgical precision. Multiple studies have demonstrated that implant positioning, limb alignment, and gap balancing remain consistently accurate from the first case onward, despite brief increases in operative time during early adoption [34, 36–38]. In a retrospective cohort study of 90 RA-TKAs and 30 conventional TKAs, Zhang et al. found greater limb alignment in the RA-TKA group, despite a brief learning curve [38]. A similar trend has been observed in robotic-assisted unicompartmental knee arthroplasty (RA-UKA). In a prospective study of 60 consecutive RA-UKA cases, Kayani et al. found that while operative times and surgical team anxiety levels followed a brief learning curve of approximately seven cases, RA-UKA demonstrated statistically superior accuracy in implant positioning and limb alignment [37]. These results highlight how robotic platforms can uphold surgical precision from the first case onward, offering immediate clinical value that complements broader institutional goals of consistency, quality, and long-term cost-effectiveness.

### Operating room workflow and personnel considerations

The successful integration of RA-TJA into the operating room demands coordinated adjustments across the entire surgical team. The well-documented learning curve of surgeons during early stages of adoption is mirrored by operating room staff. In a rapid review on robotic systems, Martinello et al. found nursing staff experience notable challenges in growing acclimated to robotic surgical systems, particularly due to the novel set-up phase, expanded surgical duties, and longer operative time [39].

To support this transition, most manufacturers provide structured training programs to help reduce variability in team performance during early implementation [40]. Additionally, the involvement of a dedicated robotic technician, responsible for system setup, calibration, and real-time intraoperative support, significantly reduces the cognitive and technical burden on surgeons and operating room staff, enabling smoother transitions between cases and enhancing intraoperative flow. Fontalis et al. specifically note the critical role of the robotic technician in troubleshooting and workflow optimization [41]. Finally, it is crucial that support from the manufacturer is well-established during implementation to ensure optimal utilization of hardware and software after purchase [42].

In short, the adoption of RA-TJA requires a teamwide adjustment in workflow, equipment handling, and interprofessional coordination. Institutions that invest in comprehensive training, clearly defined team roles, and repeatable intraoperative protocols are best positioned to translate robotic precision into scalable clinical value (Table 1).

**Table 1:** Factors contributing to cost of robotic utilization in total joint arthroplasty

Cost-Increasing Factors	Cost-Offsetting Factors
• Capital cost: $500 K–$1 M [7–9] • Annual maintenance: $40 K–150 K [7, 10] • Per-case disposables: $300–$1000 [8, 9] • Increased early operative time (12–25 min) [33–35] • Staff training + robotic technician costs [41]	• Shorter LOS [3, 8, 17, 18] • Lower 90-day complication rate [17, 25, 26] • Higher rate of home discharge [36, 43] • Lower readmission [26] • Higher alignment accuracy → fewer revisions (projected) [13, 37, 38]

*LOS* Length of Stay

### Discharge timing and recovery

When assessing the economic value of RA-TJA, it is essential to look beyond the upfront costs of acquisition and operative time. The downstream recovery trajectory, including hospital LOS, discharge disposition, and time to functional independence plays a critical role in shaping total episode-of-care expenditures and patient quality of life.

RA-TKA has been consistently associated with improved early recovery, reduced hospital LOS, and higher rates of home discharge compared to conventional approaches. A multicenter analysis of 10,296 patients by Archer et al. demonstrated that nearly 48% of RA-TKA patients were discharged within one day, compared to 39% of manual TKA patients, with a significantly higher proportion discharged directly home without skilled care (91.3% vs. 87.4%, *p* < 0.0001) [43]. Additionally, a prospective cohort study by Kayani et al. demonstrated, less postoperative pain**,** faster achievement of functional milestones, and fewer physiotherapy sessions in the RA-TKA group compared to conventional jig-based TKA [36]. Similar improvements have been observed in patients undergoing RA-THA, with numerous literature supporting significant reductions in LOS and non-home discharge with robotic implementation.

The underlying explanation for improved early recovery with RA-TJA likely involves a combination of more precise component alignment, reduced iatrogenic soft tissue injury, and lower postoperative inflammation, ultimately translating to lower pain and greater early mobilization following surgery [44]. Ultimately, these factors translate into lower opioid requirements and faster achievement of mobility milestones in the early postoperative period.

The early advantages of RA-TJA, grounded in measurable physiological changes, may be a meaningful opportunity to capture value, as even modest gains in early recovery can enhance throughput, reduce inpatient resource utilization, and improve patient satisfaction. As healthcare shifts toward bundled payments and outcome-driven reimbursement models, the early postoperative phase may emerge as a key differentiator in the cost-effectiveness of robotic platforms.

### Scheduling and throughput

Efficient scheduling is essential for integrating RA-TJA without compromising throughput. Scheduling multiple robotic procedures sequentially can minimize repeated setup and breakdown, a concept known as case clustering. This approach allows operating room teams to reduce idle time, which can ultimately match or even exceed the efficiency of manual arthroplasty [32, 45]. Meghpara et al*.* observed that early robotic cases performed in isolation showed no meaningful improvement in turnover times; however, once surgeons began clustering ≥ 3 robotic-assisted TKAs per day, a learning curve in turnover efficiency emerged, indicating that operational gains are closely tied to strategic scheduling [32]. While manual TKA generally requires less setup and transitions efficiently even when unclustered, the relative complexity of robotic workflows makes them particularly amenable to gains through repetition. In this context, RA-TJA can rival or surpass manual TKA throughput when case sequencing, team familiarity, and workflow standardization are optimized.

In addition to clustering, high-efficiency programs often rely on dedicated robotic teams, standardized checklists, and streamlined workflows to match the efficiency of manual arthroplasty [32, 46, 47]. These standardization tools help mitigate the learning curve and promote reproducibility across surgical teams and institutions. Multiple studies, including those by Putzer et al. and Shatrov et al., demonstrate that as teams gain experience, there is a significant reduction in robotic-specific setup and operative times, with improved efficiency and confidence among both surgeons and staff [46, 47]. The use of checklists and defined protocols is highlighted as a key factor in this process.

## Long-term viability and technological maturation

### Moore’s law and economies of scale

The trajectory of RA-TJA mirrors the technological pattern described by Moore’s Law, which posits that computing power doubles while costs halve over regular intervals. In the context of arthroplasty, the early adoption phase of robotics was marked by high capital costs, limited functionality, and restricted access. However, ongoing advances in hardware and software, increasing manufacturer competition, and greater market penetration have begun to drive down per-unit costs while improving the utility and precision of these systems, similar to the economic and technological maturation observed in early computing industries [48–50].

Furthermore, the diffusion of innovation framework helps contextualize the adoption pattern of robotic arthroplasty. Early adopters incur higher costs and steeper learning curves, but they pave the way for later adopters to benefit from refined workflows, broader training resources, and reduced technology prices as production scales up [7, 49, 50].

Finally, productivity gains driven by experience and workflow standardization are also well documented, with studies showing that operative times and system setup become increasingly efficient over time [15, 51, 52]. The cumulative benefits of technological innovation, scaled manufacturing, competitive pricing, and procedural refinement have collectively shifted the cost-utility curve in favor of robotic systems, reinforcing the notion that robotic arthroplasty is following a Moore’s Law-like progression toward broader accessibility and greater economic value.

### Projected trends in adoption and cost decline

The projected trajectory of RA-TJA underscores its increasing integration within orthopedic practice. Recent modeling of national inpatient data suggests that RA-KA will account for approximately 70% of all TKA procedures in the United States by 2030 [4]. This growth is not solely driven by clinical efficacy, but also by nonclinical forces such as institutional marketing strategies, patient demand, and administrative prioritization of technological differentiation [2, 49, 53].

To facilitate this widespread adoption, emerging economic models are reducing the barrier to entry. Hospitals increasingly rely on leasing and pay-per-use arrangements that allow for cost deferral and flexible financing rather than large upfront capital expenditures [49]. In high-volume health systems, robotic units are also being shared across multiple sites, further amortizing the fixed costs associated with procurement and maintenance [10, 15].

Global adoption is also expected to accelerate as unit prices decline and financing structures proliferate. Middle-income countries, where high surgical volumes often coexist with limited capital liquidity, are increasingly exploring robotic platforms, particularly when leasing models or vendor-subsidized partnerships become available [52]. While the World Health Organization has not issued specific guidance on robotic arthroplasty, its broader emphasis on surgical capacity-building in low- and middle-income countries (LMICs) creates a supportive ecosystem for future adoption. Precedents from mobile health and telemedicine demonstrate that once hardware costs decline and infrastructure stabilizes, technological catch-up in LMICs can occur rapidly [52].

### Challenges, limitations, and the need for standardization

Despite growing enthusiasm and adoption, RA-TJA faces persistent challenges that must be addressed to ensure its long-term viability and equitable integration into clinical practice. Chief among these is the lack of standardized outcome reporting across robotic platforms. Each system, whether MAKO, ROSA, CORI, or TSolution One, operates with distinct technical workflows and designs, and outcome studies often vary in endpoints, methodology, and follow-up duration. This heterogeneity has made cross-platform comparisons difficult and limits the ability to synthesize data in meta-analyses or generate consensus on best practices [54, 55]. To address these challenges in future work, each robotic system must be analyzed independently for accurate cross-platform comparisons to be made.

Additionally, major organizations such as the American Academy of Orthopaedic Surgeons (AAOS) continue to call for high-quality randomized trials and long-term outcome data to define the value proposition of robotic systems across diverse care settings. One of the most ambitious efforts is the UK Robotic Arthroplasty Clinical and Cost Effectiveness Randomised Controlled Trial for Hips (RACER-Hip) trials, which are multicenter, randomized controlled trials specifically structured to address both clinical and economic outcomes over extended follow-up periods. These trials are intended to provide robust, high-quality evidence to inform practice and policy, addressing current gaps in the literature [56, 57].

Although robotic arthroplasty holds immense promise, its continued advancement depends on standardized reporting, rigorous long-term studies, and harmonized metrics to ensure that innovation is matched by evidence, equity, and value.

## Conclusion

RA-TJA is a rapidly maturing frontier within orthopedic surgery, with growing evidence supporting its technical precision and workflow advantages. However, its value is not universally recognized. Economic viability remains highly dependent on institutional factors including surgical volume, workflow optimization, and team integration. In high-throughput environments, robotic systems can be deployed efficiently, with reduced per-case costs and favorable outcomes that align with contemporary value-based care models.

Simultaneously, the RA-TJA landscape is being reshaped by increasing market competition, emerging technologies such as AI, and broader access enabled by leasing and vendor-financed models. These forces are accelerating adoption in both high- and middle-income settings, even as definitive comparative data remain limited. The absence of standardized outcome reporting and the heterogeneity across robotic platforms continue to challenge evidence synthesis and policy development.

Ongoing multicenter randomized trials will play a pivotal role in defining the long-term clinical and economic value of robotic arthroplasty. Until such data are available, broader adoption should be guided by local infrastructure, volume capacity, and a deliberate assessment of cost–benefit tradeoffs.

## Data Availability

No datasets were generated or analysed during the current study.

## References

[1] Wang JC, Piple AS, Hill WJ et al (2022) Computer-navigated and robotic-assisted total knee arthroplasty: increasing in popularity without increasing complications. J Arthroplasty 37:2358–2364. 10.1016/j.arth.2022.06.014 35738360 10.1016/j.arth.2022.06.014

[2] Emara AK, Zhou G, Klika AK et al (2021) Robotic-arm–assisted knee arthroplasty associated with favorable in-hospital metrics and exponentially rising adoption compared with manual knee arthroplasty. J Am Acad Orthop Surg 29:e1328–e1342. 10.5435/JAAOS-D-21-00146 34037576 10.5435/JAAOS-D-21-00146

[3] Emara AK, Zhou G, Klika AK et al (2021) Is there increased value in robotic arm-assisted total hip arthroplasty?: a nationwide outcomes, trends, and projections analysis of 4,699,894 cases. Bone Joint J 103-B:1488–1496. 10.1302/0301-620X.103B9.BJJ-2020-2411.R1 34465149 10.1302/0301-620X.103B9.BJJ-2020-2411.R1

[4] Khan ST, Emara AK, Zhou G et al (2025) Robotic-assisted total knee arthroplasty in the USA: nationwide adoption trends towards 70 % by 2030. J Clin Orthop Trauma 68:103069. 10.1016/j.jcot.2025.103069 40529236 10.1016/j.jcot.2025.103069 PMC12167815

[5] Deckey DG, Rosenow CS, Verhey JT et al (2021) Robotic-assisted total knee arthroplasty improves accuracy and precision compared to conventional techniques. Bone Joint J 103-B:74–80. 10.1302/0301-620X.103B6.BJJ-2020-2003.R1 34053292 10.1302/0301-620X.103B6.BJJ-2020-2003.R1

[6] Lonner JH, Fillingham YA (2018) Pros and cons: a balanced view of robotics in knee arthroplasty. J Arthroplasty 33:2007–2013. 10.1016/j.arth.2018.03.056 29680583 10.1016/j.arth.2018.03.056

[7] Jacofsky DJ, Allen M (2016) Robotics in arthroplasty: a comprehensive review. J Arthroplasty 31:2353–2363. 10.1016/j.arth.2016.05.026 27325369 10.1016/j.arth.2016.05.026

[8] Cotter EJ, Wang J, Illgen RL (2022) Comparative cost analysis of robotic-assisted and jig-based manual primary total knee arthroplasty. J Knee Surg 35:176–184. 10.1055/s-0040-1713895 32659815 10.1055/s-0040-1713895

[9] Ezeokoli EU, John J, Gupta R et al (2023) Index surgery and ninety day re-operation cost comparison of robotic-assisted versus manual total knee arthroplasty. Intern Orthopaed 47:359–364. 10.1007/s00264-022-05674 10.1007/s00264-022-05674-w 36574020

[10] Rajan PV, Khlopas A, Klika A et al (2022) The cost-effectiveness of robotic-assisted versus manual total knee arthroplasty: a markov model-based evaluation. J Am Acad Orthop Surg 30:168–176. 10.5435/JAAOS-D-21-00309 35040808 10.5435/JAAOS-D-21-00309

[11] Tompkins GS, Sypher KS, Li H-F et al (2022) Robotic versus manual total knee arthroplasty in high volume surgeons: a comparison of cost and quality metrics. J Arthroplasty 37:S782–S789. 10.1016/j.arth.2021.12.018 34952162 10.1016/j.arth.2021.12.018

[12] Kirchner GJ, Lieber AM, Haislup B et al (2021) The cost of robot-assisted total hip arthroplasty: comparing safety and hospital charges to conventional total hip arthroplasty. J Am Acad Orthop Surg 29:609–615. 10.5435/JAAOS-D-20-00715 32991384 10.5435/JAAOS-D-20-00715

[13] Kort N, Stirling P, Pilot P, Müller JH (2022) Robot-assisted knee arthroplasty improves component positioning and alignment, but results are inconclusive on whether it improves clinical scores or reduces complications and revisions: a systematic overview of meta-analyses. Knee Surg Sports Traumatol Arthrosc 30:2639–2653. 10.1007/s00167-021-06472-4 33666686 10.1007/s00167-021-06472-4

[14] Ng N, Gaston P, Simpson PM, et al (2021) Robotic arm-assisted versus manual total hip arthroplasty: a systematic review and meta-analysis. Bone Joint J 103-B:1009–1020. 10.1302/0301-620X.103B6.BJJ-2020-1856.R1 10.1302/0301-620X.103B6.BJJ-2020-1856.R1 34058875

[15] Hua Y, Salcedo J (2022) Cost-effectiveness analysis of robotic-arm assisted total knee arthroplasty. PLoS ONE 17:e0277980. 10.1371/journal.pone.0277980 36441807 10.1371/journal.pone.0277980 PMC9704609

[16] Vermue H, Tack P, Gryson T, Victor J (2021) Can robot-assisted total knee arthroplasty be a cost-effective procedure? A markov decision analysis. Knee 29:345–352. 10.1016/j.knee.2021.02.004 33684865 10.1016/j.knee.2021.02.004

[17] Shah R, Diaz A, Phieffer L et al (2021) Robotic total knee arthroplasty: a missed opportunity for cost savings in bundled payment for care improvement initiatives? Surgery 170:134–139. 10.1016/j.surg.2020.12.046 33608146 10.1016/j.surg.2020.12.046

[18] Maldonado DR, Go CC, Kyin C et al (2021) Robotic arm-assisted total hip arthroplasty is more cost-effective than manual total hip arthroplasty: a markov model analysis. J Am Acad Orthop Surg 29:e168–e177. 10.5435/JAAOS-D-20-00498 32694323 10.5435/JAAOS-D-20-00498

[19] Seo HH, Shimizu MR, Buddhiraju A et al (2025) Utilization and reimbursements of primary total joint arthroplasty in ambulatory surgical centers: analysis of Medicare part A and B databases. J Arthroplasty 40:1445–1451. 10.1016/j.arth.2024.11.043 39586409 10.1016/j.arth.2024.11.043

[20] Habbous S, Sarma S, Lanting B et al (2025) Cost savings of outpatient versus inpatient hip and knee arthroplasty in Ontario, Canada. PLoS ONE 20:e0320255. 10.1371/journal.pone.0320255 40338887 10.1371/journal.pone.0320255 PMC12061100

[21] Eason T, Mihalko W, Toy PC (2023) Robotic-assisted total knee arthroplasty is safe in the ambulatory surgery center setting. Orthop Clin North Am 54:153–159. 10.1016/j.ocl.2022.11.001 36894288 10.1016/j.ocl.2022.11.001

[22] Kouyoumdjian P, Brichni M, Marchand P, Coulomb R (2025) Outpatient total hip arthroplasty: robotic assistance reduces 90-day postoperative events and optimizes outpatient care. Arch Orthop Trauma Surg 145:146. 10.1007/s00402-025-05767-2 39862256 10.1007/s00402-025-05767-2

[23] Lim PL, Goh GS, Bedair HS, Melnic CM (2025) Robotic versus manual total hip arthroplasty: a marginal time-driven activity-based costing analysis. J Am Acad Orthop Surg. 10.5435/JAAOS-D-24-01498 40560813 10.5435/JAAOS-D-24-01498

[24] Zalikha AK, Almsaddi T, Nham F et al (2023) Comorbidity, racial, and socioeconomic disparities in total knee and hip arthroplasty at high versus low-volume centers. J Am Acad Orthop Surg 31:e264–e270. 10.5435/JAAOS-D-22-00665 36729540 10.5435/JAAOS-D-22-00665

[25] Constantinescu DS, Costello JP, Yakkanti RR et al (2024) Lower perioperative complication rates and shorter lengths of hospital stay associated with technology-assisted total knee arthroplasty versus conventional instrumentation in primary total knee arthroplasty. J Arthroplasty 39:1512–1517. 10.1016/j.arth.2023.12.015 38103801 10.1016/j.arth.2023.12.015

[26] Howell C, Witvoet S, Scholl L et al (2025) Postoperative complications and readmission rates in robotic-assisted and manual total hip arthroplasty: a large, multi-hospital study. Med Care 63:465–471. 10.1097/mlr.0000000000002082 40497801 10.1097/MLR.0000000000002082

[27] Aggarwal VA, Sun J, Sambandam SN (2024) Outcomes following robotic assisted total knee arthroplasty compared to conventional total knee arthroplasty. Arch Orthop Trauma Surg 144:2223–2227. 10.1007/s00402-024-05231-7 38386067 10.1007/s00402-024-05231-7

[28] Siddiqi A, Mont MA, Krebs VE, Piuzzi NS (2021) Not all robotic-assisted total knee arthroplasty are the same. J Am Acad Orthop Surg 29:45–59. 10.5435/jaaos-d-20-00654 33394612 10.5435/JAAOS-D-20-00654

[29] Haas DA, Zhang X, Kaplan RS, Song Z (2019) Evaluation of economic and clinical outcomes under centers for Medicare & Medicaid Services mandatory bundled payments for joint replacements. JAMA Intern Med 179:924. 10.1001/jamainternmed.2019.0480 31157819 10.1001/jamainternmed.2019.0480 PMC6547121

[30] Blackburn CW, Du JY, Moon TJ, Marcus RE (2023) High-volume arthroplasty centers are associated with lower hospital costs when performing primary THA and TKA: a database study of 288,909 Medicare claims for procedures performed in 2019. Clin Orthop Relat Res 481:1025–1036. 10.1097/corr.0000000000002470 36342359 10.1097/CORR.0000000000002470 PMC10097563

[31] Wang KY, Margalit A, Thakkar SC et al (2021) Reimbursement for orthopaedic surgeries in commercial and public payors: a race to the bottom. J Am Acad Orthop Surg 29:e1232–e1238. 10.5435/JAAOS-D-20-01397 33750751 10.5435/JAAOS-D-20-01397

[32] Meghpara MM, Goh GS, Magnuson JA et al (2023) The ability of robot-assisted total knee arthroplasty in matching the efficiency of its conventional counterpart at an orthopaedic specialty hospital. J Arthroplasty 38:72-77.e3. 10.1016/j.arth.2022.07.024 35940350 10.1016/j.arth.2022.07.024

[33] Dragosloveanu S, Petre M-A, Capitanu BS et al (2023) Initial learning curve for robot-assisted total knee arthroplasty in a dedicated orthopedics center. JCM 12:6950. 10.3390/jcm12216950 37959414 10.3390/jcm12216950 PMC10649181

[34] Tay ML, Carter M, Zeng N et al (2022) Robotic-arm assisted total knee arthroplasty has a learning curve of 16 cases and increased operative time of 12 min. ANZ J Surg 92:2974–2979. 10.1111/ans.17975 36398352 10.1111/ans.17975 PMC9804534

[35] Marchand KB, Ehiorobo J, Mathew KK et al (2022) Learning curve of robotic-assisted total knee arthroplasty for a high-volume surgeon. J Knee Surg 35:409–415. 10.1055/s-0040-1715126 32838457 10.1055/s-0040-1715126

[36] Kayani B, Konan S, Tahmassebi J et al (2018) Robotic-arm assisted total knee arthroplasty is associated with improved early functional recovery and reduced time to hospital discharge compared with conventional jig-based total knee arthroplasty: a prospective cohort study. Bone Jt J 100-B:930–937. 10.1302/0301-620X.100B7.BJJ-2017-1449.R1 10.1302/0301-620X.100B7.BJJ-2017-1449.R1 PMC6413767 29954217

[37] Kayani B, Konan S, Pietrzak JRT, et al (2018) The learning curve associated with robotic-arm assisted unicompartmental knee arthroplasty: a prospective cohort study. Bone Joint J 100-B:1033–1042. 10.1302/0301-620X.100B8.BJJ-2018-0040.R1 10.1302/0301-620X.100B8.BJJ-2018-0040.R1 30062950

[38] Zhang H, Bai X, Wang H et al (2023) Learning curve analysis of robotic-assisted total knee arthroplasty with a Chinese surgical system. J Orthop Surg Res 18:900. 10.1186/s13018-023-04382-4 38012732 10.1186/s13018-023-04382-4 PMC10680304

[39] Martinello N, Loshak H (2020) Experiences with and Expectations of Robotic Surgical Systems: A Rapid Qualitative Review. Canadian Agency for Drugs and Technologies in Health, Ottawa (ON) 33074607

[40] Ali M, Phillips D, Kamson A et al (2022) Learning curve of robotic-assisted total knee arthroplasty for non-fellowship-trained orthopedic surgeons. Arthroplasty Today 13:194–198. 10.1016/j.artd.2021.10.020 35118183 10.1016/j.artd.2021.10.020 PMC8791856

[41] Fontalis A, Hansjee S, Giebaly DE et al (2024) Troubleshooting robotics during total hip and knee arthroplasty. Orthop Clin North Am 55:33–48. 10.1016/j.ocl.2023.06.004 37980102 10.1016/j.ocl.2023.06.004

[42] Saber AY, Marappa-Ganeshan R, Mabrouk A (2025) Robotic-Assisted Total Knee Arthroplasty. In: StatPearls. StatPearls Publishing, Treasure Island (FL) 33232066

[43] Archer A, Salem HS, Coppolecchia A, Mont MA (2023) Lengths of stay and discharge dispositions after total knee arthroplasty: a comparison of robotic-assisted and manual techniques. J Knee Surg 36:404–410. 10.1055/s-0041-1735280 34610638 10.1055/s-0041-1735280

[44] Fontalis A, Kayani B, Asokan A et al (2022) Inflammatory response in robotic-arm-assisted versus conventional jig-based TKA and the correlation with early functional outcomes: results of a prospective randomized controlled trial. J Bone Joint Surg 104:1905–1914. 10.2106/JBJS.22.00167 36074816 10.2106/JBJS.22.00167

[45] Witvoet S, De Massari D, Shi S, Chen AF (2023) Leveraging large, real-world data through machine-learning to increase efficiency in robotic-assisted total knee arthroplasty. Knee Surg Sports Traumatol Arthrosc 31:3160–3171. 10.1007/s00167-023-07314-1 36650339 10.1007/s00167-023-07314-1

[46] Shatrov J, Foissey C, Batailler C et al (2023) How long does image based robotic total knee arthroplasty take during the learning phase? Analysis of the key steps from the first fifty cases. Intern Orthopaed 47:437–446. 10.1007/s00264-022-05618-4 10.1007/s00264-022-05618-4 36355082

[47] Putzer D, Schroeder L, Wassilew G et al (2024) Early learning curve in robotic-assisted total knee arthroplasty: a single-center experience. JCM 13:7253. 10.3390/jcm13237253 39685712 10.3390/jcm13237253 PMC11641829

[48] Parsley BS (2018) Robotics in orthopedics: a brave new world. J Arthroplasty 33:2355–2357. 10.1016/j.arth.2018.02.032 29605151 10.1016/j.arth.2018.02.032

[49] Mont MA, Khlopas A, Chughtai M et al (2018) Value proposition of robotic total knee arthroplasty: what can robotic technology deliver in 2018 and beyond? Expert Rev Med Devices 15:619–630. 10.1080/17434440.2018.1515011 30134738 10.1080/17434440.2018.1515011

[50] Boylan M, Suchman K, Vigdorchik J et al (2018) Technology-assisted hip and knee arthroplasties: an analysis of utilization trends. J Arthroplasty 33:1019–1023. 10.1016/j.arth.2017.11.033 29290333 10.1016/j.arth.2017.11.033

[51] Innocenti B, Bori E (2021) Robotics in orthopaedic surgery: why, what and how? Arch Orthop Trauma Surg 141:2035–2042. 10.1007/s00402-021-04046-0 34255170 10.1007/s00402-021-04046-0

[52] Peng Y, Liu Y, Lai S et al (2023) Global trends and prospects in health economics of robotic surgery: a bibliometric analysis. Int J Surg. 10.1097/JS9.0000000000000720 37720937 10.1097/JS9.0000000000000720 PMC10720792

[53] Sherman WF, Wu VJ (2020) Robotic surgery in total joint arthroplasty: a survey of the AAHKS membership to understand the utilization, motivations, and perceptions of total joint surgeons. J Arthroplasty 35:3474-3481.e2. 10.1016/j.arth.2020.06.072 32731999 10.1016/j.arth.2020.06.072

[54] Vermue H, Batailler C, Monk P et al (2022) The evolution of robotic systems for total knee arthroplasty, each system must be assessed for its own value: a systematic review of clinical evidence and meta-analysis. Arch Orthop Trauma Surg 143:3369–3381. 10.1007/s00402-022-04632-w 36153769 10.1007/s00402-022-04632-w

[55] Elliott J, Shatrov J, Fritsch B, Parker D (2021) Robotic-assisted knee arthroplasty: an evolution in progress. A concise review of the available systems and the data supporting them. Arch Orthop Trauma Surg 141:2099–2117. 10.1007/s00402-021-04134-1 34491411 10.1007/s00402-021-04134-1

[56] Griffin J, Davis ET, Parsons H et al (2023) UK robotic arthroplasty clinical and cost effectiveness randomised controlled trial for hips (RACER-Hip): a study protocol. BMJ Open 13:e079328. 10.1136/bmjopen-2023-079328 37852762 10.1136/bmjopen-2023-079328 PMC10603453

[57] Griffin J, Davis ET, Parsons H et al (2023) Robotic arthroplasty clinical and cost effectiveness randomised controlled trial (RACER-knee): a study protocol. BMJ Open 13:e068255. 10.1136/bmjopen-2022-068255 37295832 10.1136/bmjopen-2022-068255 PMC10277111

